# The phenotypic spectrum of *WWOX***-**related disorders: 20 additional cases of WOREE syndrome and review of the literature

**DOI:** 10.1038/s41436-018-0339-3

**Published:** 2018-10-25

**Authors:** Juliette Piard, Lara Hawkes, Mathieu Milh, Laurent Villard, Renato Borgatti, Romina Romaniello, Melanie Fradin, Yline Capri, Delphine Héron, Marie-Christine Nougues, Caroline Nava, Oana Tarta Arsene, Debbie Shears, John Taylor, Alistair Pagnamenta, Jenny C Taylor, Yoshimi Sogawa, Diana Johnson, Helen Firth, Pradeep Vasudevan, Gabriela Jones, Marie-Ange Nguyen-Morel, Tiffany Busa, Agathe Roubertie, Myrthe van den Born, Elise Brischoux-Boucher, Michel Koenig, Cyril Mignot, DDD Study, Usha Kini, Christophe Philippe

**Affiliations:** 1 Centre de Génétique Humaine, Université de Franche-Comté, CHU Besançon, Besançon, France; 2 Oxford Centre for Genomic Medicine, Oxford University Hospitals NHS Foundation Trust, Oxford, UK; 3 Aix Marseille Univ, Inserm, MMG, Marseille, France; 4 Pediatric Neurology, La Timone Children’s Hospital, AP-HM, Marseille, France; 5 Medical Genetics, La Timone Children’s Hospital, AP-HM, Marseille, France; 6 Neuropsychiatry and Neurorehabilitation Unit, Scientific Institute IRCCS Eugenio Medea, Bosisio Parini, Italy; 7 Service de Génétique, CLAD Ouest, CHU Rennes, Rennes, France; 8 Département de Génétique, Hôpital Robert Debré, APHP Paris, Paris, France; 9 APHP, Département de Génétique, Centre de Référence Déficiences Intellectuelles de Causes Rares, Groupe Hospitalier Pitié Salpêtrière et GHUEP Hôpital Trousseau, Sorbonne Université, GRC “Déficience Intellectuelle et Autisme”, Paris, France; 10 Neuropédiatrie et Unité d’électrophysiologie clinique, Centre de Référence des Maladies Neuromusculaires de l’EST parisien et DHU I2B, Hôpital d’Enfants Armand Trousseau, Paris, France; 11 Département de Génétique, Sorbonne Universités, Institut du Cerveau et de la Moelle épinière, ICM, Inserm U1127, CNRS UMR 7225, APHP, Hôpital de la Pitié Salpêtrière, Paris, France; 12 Pediatric Neurology Clinic, “Alexandru Obregia” Clinical Psychiatry Hospital, “Carol Davila” University of Medicine, Bucharest, Romania; 13 Oxford Medical Genetics Laboratories, Oxford University Hospitals NHS Foundation Trust, Churchill Hospital, Oxford, UK; 14 NIHR Oxford Biomedical Research Centre, Wellcome Centre for Human Genetics, University of Oxford, Oxford, UK; 15 Division of Pediatric Neurology, Children’s Hospital of Pittsburgh of UPMC, Pittsburgh, PA USA; 16 Department of Clinical Genetics, Sheffield Children’s NHS Trust, Sheffield, United Kingdom; 17 Department of Clinical Genetics, Cambridge University Hospitals NHS Foundation Trust, Cambridge, UK; 18 Wellcome Trust Sanger Institute, Wellcome Genome Campus, Hinxton, UK; 19 Clinical Genetics Department, University Hospitals Leicester NHS Trust, Leicester, UK; 20 Service de Neurologie pédiatrique, Hopital Couple Enfant, CHU Grenoble Alpes, Grenoble, France; 21 Département de Neuropédiatrie, Centre Hospitalier Universitaire de Montpellier, INSERM U 1051, Institut des Neurosciences de Montpellier, Montpellier, France; 22 Department for Clinical Genetics, Erasmus MC, Rotterdam, Netherlands; 23 EA7402 Institut Universitaire de Recherche Clinique, and Laboratoire de Génétique Moléculaire, CHU and Université de Montpellier, Montpellier, France; 24 Laboratoire de génétique, Innovations en diagnostic génomique des maladies rares, Plateau Technique de Biologie, CHU Dijon, Dijon, France; 25 INSERM 1231, LNC UMR1231 GAD, Burgundy University, Dijon, France

**Keywords:** *WWOX*, encephalopathy, epilepsy

## Abstract

**Purpose:**

Germline *WWOX* pathogenic variants have been associated with disorder of sex differentiation (DSD), spinocerebellar ataxia (SCA), and *WWOX*-related epileptic encephalopathy (WOREE syndrome). We review clinical and molecular data on *WWOX*-related disorders, further describing WOREE syndrome and phenotype/genotype correlations.

**Methods:**

We report clinical and molecular findings in 20 additional patients from 18 unrelated families with WOREE syndrome and biallelic pathogenic variants in the *WWOX* gene. Different molecular screening approaches were used (quantitative polymerase chain reaction/multiplex ligation-dependent probe amplification [qPCR/MLPA], array comparative genomic hybridization [array-CGH], Sanger sequencing, epilepsy gene panel, exome sequencing), genome sequencing.

**Results:**

Two copy-number variations (CNVs) or two single-nucleotide variations (SNVs) were found respectively in four and nine families, with compound heterozygosity for one SNV and one CNV in five families. Eight novel missense pathogenic variants have been described. By aggregating our patients with all cases reported in the literature, 37 patients from 27 families with WOREE syndrome are known. This review suggests WOREE syndrome is a very severe epileptic encephalopathy characterized by absence of language development and acquisition of walking, early-onset drug-resistant seizures, ophthalmological involvement, and a high likelihood of premature death. The most severe clinical presentation seems to be associated with null genotypes.

**Conclusion:**

Germline pathogenic variants in *WWOX* are clearly associated with a severe early-onset epileptic encephalopathy. We report here the largest cohort of individuals with WOREE syndrome.

## Introduction

*WWOX* (WW domain-containing oxidoreductase) is located on chromosome 16q23.1-q23.2 and was first implicated in cancer. It crosses the second most common fragile site (FRA16D) in the human genome. Somatic sequence variants in *WWOX* were found in many types of neoplasia and *WWOX* is recognized as an important tumor suppressor gene.^1^ More recently, germline pathogenic variants in *WWOX* were implicated in constitutional diseases and three different WWOX-related phenotypes were described: disorder of sex differentiation (DSD), spinocerebellar ataxia (SCA), and epileptic encephalopathy. A heterozygous in-frame deletion of exons 6 to 8 in *WWOX* was first associated with DSD in a young male patient.^2^ Comprehensive genetic analyses by array comparative genomic hybridization (array-CGH) or exome sequencing (ES) identified *WWOX* pathogenic variants in autosomal recessive neurological diseases. Homozygous missense pathogenic variants in *WWOX* were found in two consanguineous families with spinocerebellar ataxia type 12 (SCAR12) associated with epilepsy and intellectual disabilty.^3^ Biallelic pathogenic variants in *WWOX* were subsequently implicated in a form of autosomal recessive infantile epileptic encephalopathy called *WWOX*-related epileptic encephalopathy (WOREE) syndrome.^4–9^

We report here 20 additional patients with biallelic pathogenic variants in the *WWOX* gene associated with severe early-onset encephalopathy. After a literature review, we define the main clinical features of the *WWOX*-related disorders and discuss genotype–phenotype correlations.

## Materials and methods

### Patients

Individuals with *WWOX* biallelic pathogenic variants and epileptic encephalopathy were collected from France, England, Italy, the Netherlands, Romania, and the United States. Copy-number variations (CNVs) were identified by multiplex ligation-dependent probe amplification (MLPA) (4 patients) or array-CGH (4 patients). Single-nucleotide variations (SNVs) were identified by targeted *WWOX* Sanger sequencing (2 patients), epilepsy gene panel (2 patients), exome sequencing (ES) 9 patients), or by GS (genome sequencing), 1 patient. All variations identified by epilepsy gene panel, ES, and array-CGH were confirmed by Sanger sequencing and MLPA/quantitative polymerase chain reaction (qPCR) respectively. In all cases, parents were tested by targeted Sanger sequencing, MLPA, or qPCR to confirm that the variations found in the proband were in *trans*. Upon identification of two pathogenic or likely pathogenic variants in *WWOX*, the referring clinicians were contacted and the clinical data collected (Supplemental Table 1). Written informed consent was obtained for genetic testing and use of photographs (if applicable).

### Methods

#### Array-CGH

CNVs were detected using the Human Genome Microarray CGH 180 K, from Agilent® according to the manufacturer’s protocol (Agilent Technologies, Santa Clara, CA) and different Illumina single-nucleotide polymorphism (SNP) arrays (Human Omni 1, Omni 2.5, and Omni 5 BeadChip arrays). Data of the CGH were processed with Feature Extraction (v. 12.0.1.1) software and the results were analyzed with CytoGenomics (v. 3.02.11) software (Agilent®). Mapping data were analyzed on the human genome sequence using Ensembl (Hg19). (ref. ^10^). CNVs were assessed in BENCH Lab CNV (v5.1.1). CNV changes identified by microarray in the affected child analysis were confirmed by quantitative qPCR in the proband and both parents. Real-time PCR reactions were performed in the LightCycler 480 system (Roche) using the SYBR Green I Master Kit (Eurogentec, Seraing, Belgium) with 2 mL of complementary DNA (cDNA) and 200 nM of each primer. Each reaction was performed in triplicate.^11^

#### MLPA

Large rearrangement screening of the *WWOX* gene or validation of CNVs involving all or part of the WWOX locus identified by array-CGH was performed by MLPA (MRC-Holland). We designed synthetic probes for all nine exons of the *WWOX* gene not covered by any probe mix available from MRC-Holland at the time of this study. DNA sequences of MLPA probes used in this study are available upon request. ABIF files were analyzed by using the Coffalyser.Net, a free MLPA analysis software developed by MRC-Holland.

#### Epilepsy gene panel

For patients 6 and 7, a gene panel comprising 90 genes involved in epilepsy was performed. The genes were selected based on previous literature and OMIM databases. A library of all coding exons and intron–exon boundaries was prepared using a SeqCap EZ Library (Roche-NimbleGen, USA) following the manufacturer’s instructions. Gene panel sequencing was performed on a MiSeq (Illumina Inc., CA). Sequence alignment was performed by Genodiag (Paris, France) with Burrows–Wheeler Aligner (BWA) 0.7.12, picard-tools-1.121, GATK-3.5 (Indel realignment, base recalibration). Then, the variant calling and annotations were made with Genome Analysis Toolkit (GATK)-3.5 (HaplotypeCaller) and SNPEff-4.2 with additional information from gnomAD, ClinVar, and the Human Gene Mutation Database (HGMD).

#### ES

Solo or trio-based ES approaches were performed. One microgram of genomic DNA extracted from blood leukocytes was used for targeted exon enrichment with the SureSelect Human All Exon V4 kit (Agilent) on a HiSeq 2000 instrument (Illumina) according to the manufacturer’s recommendations for paired-end 76-bp reads. Raw data were processed as previously described.^12^ AM files were aligned to a human genome reference sequence (GRCh37/hg19) using BWA v0.6.7 and potential duplicate paired-end reads were removed by Picard 1.109. Indel realignment and base quality score recalibration were conducted with GATK v3.3-0. Variations with a quality score >30 and alignment quality score >20 were annotated with SeattleSeq SNP Annotation.^13^ Rare variations present at a frequency above 1% in dbSNP 138 and the National Heart, Lung, and Blood Institute (NHLBI) GO Exome Sequencing Project or present from local exomes of unaffected individuals were excluded. Variation prioritization focused on de novo heterozygous, compound heterozygous, or hemizygous variants affecting the coding sequence (missense, nonsense, and splice-site variants and coding indels). Candidate variants were then inspected with the Integrative Genomics Viewer.^14^ Candidate variants were confirmed by means of Sanger sequencing.

#### GS

A Solo GS approach was performed. One microgram of genomic DNA was prepared using an Illumina TruSeq DNA PCR-free library preparation kit and then sequenced on a HiSeq 2000 instrument (Illumina) according to the manufacturer’s recommendations. Sequence alignment and variant calling were performed within BaseSpace (basespace.illumina.com) using ISAAC Whole Genome Sequencing v3. Sequence reads were aligned to the human genome reference sequence (GRCh37/hg19). Structural variant analysis was performed using BreakDancer^15^ and copy number variation analysis was undertaken using BIC-seq software.^16^ Rare variations present at a frequency above 1% in dbSNP 138 and the NHLBI GO Exome Sequencing Project were excluded. Variants were restricted to a targeted epileptic encephalopathy-related gene panel, which included WWOX (a list of targeted genes is available on request). Candidate variants were confirmed by Sanger sequencing and testing parental samples confirmed the WWOX variants were on separate alleles.

#### Sanger sequencing

We screened for SNVs the entire coding sequence plus exon–intron boundaries by direct sequencing of PCR products. Exons 1 to 9 of the *WWOX* gene were PCR amplified (primer sequences available upon request). Primers were modified by the addition of either M13F (5’-tgtaaaacgacggccagt-3’) or M13R (5’-caggaaacagtcatgacc) sequences at their 5’ end. The coding sequence was screened by direct DNA sequencing with M13F and M13R primers as described earlier.^17^ Sequences were automatically analyzed with the Seqscape 2.5 software (Applied Biosystems).

#### Description of sequence variants

Sequence variants in the *WWOX* gene are numbered starting from the first base of the ATG codon, numbering based on the 9 coding exons reference sequence NM_016373.2. For CNVs detected by array-CGH, the distances from the 16p telomere are derived from the National Center for Biotechnology Information (NCBI) Genome Browser (build 37) (ref. ^18^). Description of the sequence (Human Genome Variation Society)^19^ was done with the assistance of Mutalyser 2.0.26 with the Name Checker DNA tool.

## Results

We report clinical and molecular findings in 20 patients with severe early-onset epileptic encephalopathy and biallelic pathogenic variants affecting the WWOX locus.

### Molecular findings

Biallelic genetic anomalies were compound heterozygous in 14 index patients and homozygous in the others with genetically related parents (Supplemental Table 1). The *WWOX* genotypes were composed of two CNVs or two SNVs in 4 and 9 families respectively. In 5 families, a compound heterozygous status with one SNV and one CNV has been revealed through a combination of two molecular screening approaches. Two patients (P2 and P4) were found to carry two different CNVs, each affecting a single exon. After array-CGH, P4 was initially described as heterozygous for a deletion affecting exons 5 to 7 of *WWOX*. Because the clinical phenotype in P4 is very reminiscent of WOREE syndrome, we decided to perform a complementary targeted *WWOX* screening. The correct compound heterozygous status with two single exon deletions in P4 was only determined after MLPA analysis. Both CNVs in P2 were characterized by a first-line MLPA analysis.

We identified eight new missense variations in *WWOX*, two of which were not located in a known functional domain: p.(Glu17Lys) and p.(Thr358Ile) (Supplemental Table 2). Four missense variations were recurrent: p.(Gln230Pro) was found in four families, p.(Gly137Glu) in three families, and p.(Ser318Leu) and p.(Pro47Arg) in two families. All missense variations identified in this study but one (p.[Ser318Leu]) were classified as probably damaging by the PolyPhen-2 prediction tool. None of the eight missense variants reported for the first time in this study are present in a homozygous state in gnomAD^20^ (Supplemental Table 2).

### Demographic data

Twenty patients belonging to 18 unrelated families were identified with biallelic *WWOX* pathogenic variants. Eight were male (40%) and 12 were female (60%). The median age at last follow-up was 52 months (min: 3 months; max: 17 years). The majority of patients were unrelated except for two pairs of siblings (P13 and P14; P15 and P16). Among the 18 families, 5 were consanguineous (28%).

### Prenatal and neonatal abnormalities

Prenatal features were observed in 5 of 20 patients (25%). Anomalies described included decreased fetal movement for three cases (with talipes in one), macrosomia, and unilateral clubfoot. In the neonatal period, hands and feet anomalies (not seen by ultrasound follow-up) were reported in three more patients (clenched fists, flexed hands, and clubfeet). Other anomalies were feeding difficulties in two patients, hypotonia, lip smacking, and jaundice (each in one case). One patient required resuscitation.

### General clinical data

Mean height was –1,1 SD (standard deviation) with only one patient under 3 SD (1/17; 6%). Mean occipitofrontal circumference (OFC) was –1,4 SD and 4 of 20 (20%) patients had microcephaly (defined by OFC ≤ –3 SD). Facial dysmorphism was reported in 12 of 20 (60%) individuals. Scoliosis or kyphosis was present in 13 of 20 (65%) patients. Feeding difficulties were observed in most of the patients and 13/19 (70%) required a feeding tube. Respiratory problems including respiratory failure, asthma, recurrent infections, irregular breathing pattern, or aspiration pneumonia were reported in 8 of 20 (40%) patients. Hearing loss and cleft palate were each reported in a single patient. Premature death (i.e., before 3 years) occurred in 8 of 20 patients (40%) with mean age at death of 40 months (range: 6 months to 8 years 11 months).

### Neurodevelopmental data

All patients had profound developmental delay and were not able to either sit or walk. They had no language. Poor spontaneous movements were reported in 17 of 19 (89%). Hypotonia (mostly axial) was observed in 15 of 20 (75%) patients and hypertonia (mostly distal) in 15 of 18 (83%). Various movement disorders were present in 6 of 18 (33%) cases.

All patients developed seizures (20/20; 100%). Mean age of onset was 1.6 months ranging from day 1 to 7 months. Drug resistance was reported in 19 of 20 (95%) patients. Where frequency of seizures was reported, all patients had daily seizures (up to >30 per day). Seizures were of varying types and were either focal or generalized. Few patients had febrile seizures (2/17; 12%) and 4 of 17 (23%) had status epilepticus. A defined epileptic syndrome was documented in 7 of 19 (37%) patients. Five developed West syndrome (5/19; 26%) and two developed Lennox–Gastaut syndrome (2/19; 11%).

Brain magnetic resonance image (MRI) was abnormal in 80% (16/20) of our patients; corpus callosum hypoplasia (15/20; 75%) and progressive cerebral atrophy (11/20; 55%) being the most prominent anomalies. White matter hypersignal (2/20) and delayed myelination (1/20) were occasionally reported.

### Ophthalmological features

Of 20 patients, 16 (75%) had no eye contact or poor eye contact. Fundus oculi was altered in 9/19 patients (47%) and electrodiagnostic tests were abnormal in 5/11 (45%) patients. Retinal dystrophy was diagnosed in 2/19 (10%) patients and optic nerve anomalies in 10/19 (53%).

## Discussion

**Fig. 1:**
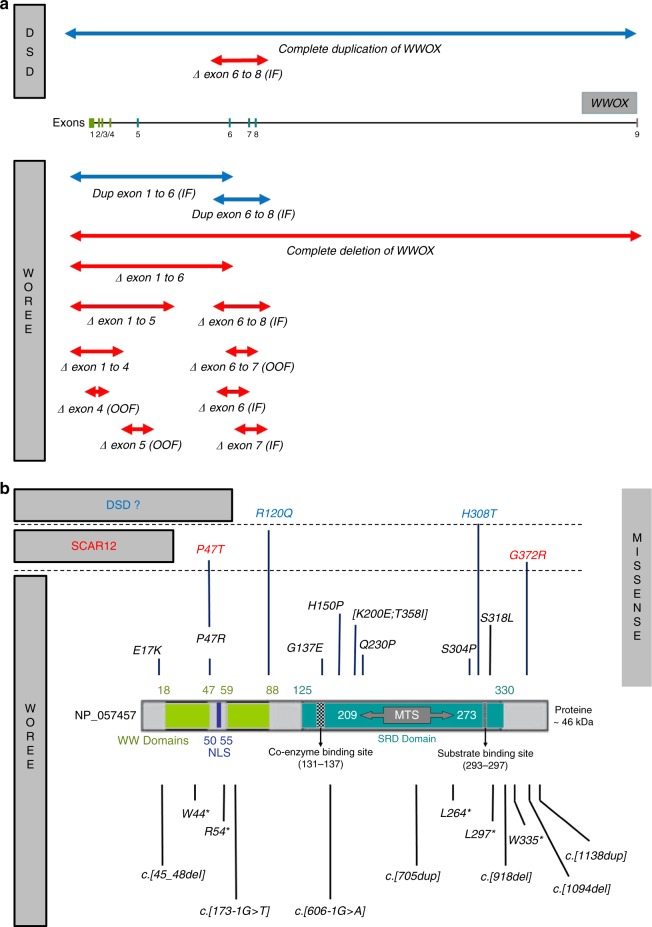
***WWOX*** **germline pathogenic variants identified in patients with constitutional** ***WWOX*-related disorders (SCAR12, WOREE syndrome, and DSD).** **a** Copy-number variations (*n* = 12). *DSD* disorder of sex development, *IF* in frame, *OOF* out of frame, *WOREE* *WWOX*-related epileptic encephalopathy. **b** Single-nucleotide variations (*n* = 23). *MTS* mitochondrial targeting sequence, *NLS* nuclear localization signal, *SRD* short-chain dehydrogenase reductase.

*WWOX* is located on the long arm of chromosome 16 in a known fragile site. It is composed of nine exons and is >1 Mb in size.^1^ The canonical transcript (ENST00000566780.5, NM_016373.3) encodes a 414–amino acid (aa) ubiquitous protein highly expressed in the prostate, gonads, breast, lung, endocrine tissues, and brain.^21,22^ Alternative splicing of *WWOX* during precursor messenger RNA (pre-mRNA) processing generates eight transcripts that have been observed only in cancers; whether every aberrant *WWOX* mRNA is translated into protein is unknown.^23^ The WW domain-containing oxidoreductase is the only protein that couples two distinct domains with high homologies with both the WW domain family and SRD superfamily. WWOX harbors two WW domains at the amino terminus, a nuclear localization signal (NLS) motif and a C-terminal short-chain dehydrogenase reductase (SDR) domain (Fig. 1b). A mitochondrial targeting sequence (MTS) motif has also been mapped within the SDR domain between aa 209 and aa 273 (ref. ^24^). WWOX also contains both a coenzyme, NAD(H) or NADP(h) binding site (GANSGIG, aa 131–137), and a potential substrate binding site (YNRSK, aa 293–297) (ref. ^1^). The C-terminal domain of WWOX (aa 125–414) is similar to a MAPT interaction region (http://www.uniprot.org/uniprot/Q9NZC7).

*WWOX* is tolerant to missense variations with a missense constraint metric *Z* = –3.19 (Exome Aggregation Consortium).^20^ Many heterozygous CNVs affecting all or part of this gene are observed in healthy control samples (Database of Genomic Variants).^25^ The high number of pathogenic/probably pathogenic variations in public databases is not surprising considering an autosomal recessive mode of inheritance for *WWOX*-related encephalopathy.

*WWOX* is expressed in mouse developing nervous system including cerebral cortex and cerebellum from in utero stages to adulthood.^26^ Animal models demonstrated epilepsy and ataxia caused by *WWOX* loss,^3,27^ suggesting a neurodegenerative process. In a detailed review of the literature, Aldaz and collaborators clearly demonstrated that *WWOX* is at the crossroads of cancer, metabolic syndrome, and encephalopathy.^28^

Clinical and molecular data for all patients from the literature with either SCAR12 or WOREE phenotypes are described in Supplemental Table 3 (refs. ^3–8,29,30^). Two additional patients, sisters from a consanguineous Arab family with a *WWOX* homozygous variant (c.1228G>T; p.Gly410Cys), were also reported by Alkhateeb et al. in 2016 (ref. ^9^), but there is doubt as to the pathogenicity of the variant identified in these cases. Both presented with delayed speech and one had epilepsy from 2 years of age. The clinical presentation in this family is very mild as compared with the WOREE syndrome observed in patients with biallelic germline pathogenic variants in *WWOX*. This glycine to cysteine change at position 410 affects an amino acid at the very end of the carboxyl terminus side of the protein. Additionally this amino acid change is present in homozygous form in two individuals in the gnomAD data set. Although in silico predictions are in favor of the pathogenicity of this missense pathogenic variant (possibly damaging by PolyPhen-2), its causal role is questionable in this family. We have therefore decided to exclude these patients from our literature review.

We report here on the largest cohort of individuals with a WOREE phenotype consisting of 20 additional patients with biallelic pathogenic variants in *WWOX* inherited from healthy parents (Supplemental Table 1). Molecular diagnoses were obtained by a combination of different techniques (array-CGH, qPCR/MLPA, targeted Sanger sequencing, ES and WGS). By aggregating our patients with all cases reported so far in the literature, 37 patients from 27 families with a WOREE syndrome and biallelic *WWOX* pathogenic variants are known. A total of 29 different pathogenic/likely pathogenic alleles (10 CNVs and 19 SNVs) have been identified (Fig. 1a, b). Introns 5 and 8 of the *WWOX* gene are very large with a size of 220 and 780 Kb respectively. They cover 90% of the *WWOX* genomic sequence and contain translocation breakpoints for multiple myeloma,^28^ in accordance with the fact that large intronic regions are often recombinogenic and prone to chromosomal breakpoints. Accordingly, duplication (one allele) and deletion (five alleles) encompassing exons 6 to 8 are the most frequent pathogenic CNVs observed in patients with *WWOX*-related disorders (Supplemental Tables 1 and 3). All patients from consanguineous families (10/27; 37%) were found to be homozygous for the same deleterious allele, a common occurrence in autosomal recessive disorders with a low prevalence. The high proportion of families with a genotype comprising one or two pathogenic CNVs (14/27; 52%) highlights the need for a quantitative approach for screening the *WWOX* locus in patients with severe central nervous system disorders. In patients heterozygous for a CNV affecting several exons, it is important to search for (1) a possible compound heterozygous genotype for two small CNVs and the need for a second quantitative analysis by MLPA or qPCR in both parents and (2) a SNV on the second allele by targeted Sanger sequencing. In the same way, when ES or targeted gene panel sequencing is performed as a first-line investigation, the search for CNVs is essential in this large gene prone to deletions and duplications, involving all or part of its coding sequences.

In this study we report eight new missense pathogenic variants (Supplemental Table 2). Among exome and targeted gene panel high-throughput sequencing results, *WWOX* missense variants are numerous. Their pathogenicity is difficult to establish because WWOX has numerous functions and protein partners. We followed American College of Medical Genetics and Genomics (ACMG) guidelines^31^ for interpreting and annotating missense variations in *WWOX* data to classify them as pathogenic, likely pathogenic, variants of unknown significance (VUS), likely benign, or benign. To aid our variant interpretation process, we used an openly available online tool (http://www.medschool.umaryland.edu/Genetic_Variant_Interpretation_Tool1.html/) to efficiently classify variants based on the evidence categories outlined by ACMG guidelines.^31^ Four aa changes (p.[Gly137Glu], p.[His150Pro], p.[Gln230Pro], p.[Ser318Leu]) are associated in *trans* with null variants in a gene where loss of function is a known disease mechanism. All missense variants identified in individuals with *WWOX*-related encephalopathy (11 aa changes in 12 families, Supplemental Table 2) are predicted to be disease-causing by a combination of web-based softwares. More importantly, none are present in a homozygous state in gnomAD.^20^ All missense variants are classified as pathogenic or likely pathogenic according to the ACMG recommendations. One patient (P19) is a carrier of three different missense pathogenic variants: p.[Lys200Glu;Thr358Ile];[Gln230Pro] (Supplemental Table 1). The p.(Lys200Glu) and p.(Thr358Ile) are located in *cis*; both amino acid changes are predicted to be likely pathogenic although the threonine at position 358 is not located in a known functional domain. It is not possible to determine whether one or both of these variants are the disease-causing missense variant in P19. In most cases reported here and published in the medical literature, *WWOX* sequence variations are described at the basic DNA level. All missense pathogenic variants but one (P3; p.[Ser318Leu]) are interpreted based on in silico studies without experimental evidence (no RNA or protein sequence analyzed). In P3, total RNA analysis by Sanger sequencing of reverse transcription PCR (RT-PCR) products failed to detect any splicing anomaly in leukocytes. RNA studies were not performed for other patients because appropriate blood samples were not available at the time of the molecular diagnosis. Exonic SNVs could affect physiological acceptor/donor splice sites, but also exonic splicing enhancer (ESE) and exonic splicing silencer (ESS). Moreover, the activation of cryptic splice sites by SNVs is a well-understood pathological mechanism in many genetic disorders; a good example of this is Hutchinson–Gilford progeria syndrome.^32^ Missense variants could also impact on mRNA stability.^33^ Up to 10% of known disease-associated missense variants, but only 3% of common SNPs, alter pre-mRNA splicing.^33^ It is likely that a fraction of predicted missense variants identified in patients with *WWOX*-related encephalopathy results in loss of expression due to abnormal splicing. This is all the more probable for a gene with pathogenic loss-of-function alleles. For missense pathogenic variants in the SRD domain, the absence of identified substrates makes it difficult to evaluate their impact on WWOX’s catalytic activity. Characterization of knock-in animal models such as zebrafish would give clues about the pathogenicity of *WWOX* missense variants.

In one patient not included in this study, a molecular diagnosis of *WWOX*-related encephalopathy was initially suspected in front of a biallelic genotype consisting of two SNVs in *trans*: a nonsense (ENST00000566780.5:p.Gln72*) and a missense variant (ENST00000402655:c.600T>A, p.Ser200Arg). The aa change at position 200 only affects a non-RefSeq WWOX protein of 311 residues. So far, all pathogenic aa changes have been described according to the canonical WWOX protein (414 aa), which results from the translation of a 9-exon transcript (ENST00000566780.5, isoform 1). It is unknown whether the WWOX protein isoform composed of 311 residues is relevant for WWOX-related disorders. Additionally, the phenotype in this patient was mild compared with the WOREE syndrome. For these reasons, we decided not to consider this genotype as causative for the encephalopathy in this patient.

### WWOX, cancer, and lung disease?

*WWOX* is a tumor suppressor gene involved in the modulation of cancer-related pathways via protein–protein interactions between the WW domains and various oncogenic proteins. Loss of WWOX protein was found in multiple neoplasias.^23^ Osteosarcomas and lung carcinomas are observed in Wwox-/- mice^22^ and tumorigenesis is one of the consequences of a Wwox hypomorphic genotype.^34^ A functional loss of copy CNV (67048) in *WWOX* was significantly associated with an increased cancer risk in Chinese populations.^35–37^ More recently, loss of WWOX expression in mice lung was shown to cause neutrophilic inflammation.^38^ Surprisingly, increase in cancer cases was never reported in either patients with biallelic *WWOX* germline pathogenic variants or their heterozygous parents. The severity of WOREE syndrome with a high frequency of premature death in the first years of life could explain the absence of any cancers. This is not a convincing explanation for patients heterozygous for a *WWOX* null allele. Ablation of Wwox is not tumorigenic in all knockout animal models; this putative tumor suppressor gene is more likely to be of relevance in tumor progression rather than initiation during oncogenesis. This study confirms that germline *WWOX* alterations are not driver pathogenic variants important for cancer initiation and/or progression.

### *WWOX* and DSD

*WWOX* is highly expressed in hormonally regulated tissues such as the pituitary, testis, and ovary, and is presumed to play a role in gonadotropin or sex-steroid biosynthesis.^1,39^ The SRD enzymatic domain in WWOX is thought to have a role in steroid metabolism with as yet unidentified substrates. These observations and the description of two different *WWOX* knockout mouse models with gonadal abnormalities^34,39^ clearly support a role for *WWOX* in gonad development.

The first germline rearrangement of *WWOX* in humans was associated with a 46, XY DSD.^2^ The patient had bilateral undifferentiated gonadal tissue and immature testes. He was heterozygous for a deletion of exons 6 to 8 inherited from his mother who had a personal history of late menarche. This CNV is predicted to result in an in-frame deletion, causing shortened product with the SRD domain largely missing. More recently, three heterozygous VUS in the *WWOX* gene were identified by ES in patients with DSD (Fig. 1) (ref. ^40^). To our knowledge, DSD has not been reported in heterozygous parents of patients with WOREE syndrome and we believe that the implication of heterozygous SNVs/CNVs in the molecular etiology of DSD is questionable. Testicular atrophy, reduced fertility, gonadal abnormalities, Leydig cell dysfunction, and impaired mammary development are part of the phenotypes observed in several animal models of WWOX ablation.^28^ To our knowledge, DSD is not reported in heterozygous knockout animal models. However, a possibility remains that a milder phenotype could be identified after careful evaluation of sex development and gonadal function in patients heterozygous for a *WWOX* pathogenic variant.

### *SCAR12* phenotype

In 2014, homozygous missense pathogenic variants in *WWOX* were identified by ES in six patients from two consanguineous families with autosomal recessive spinocerebellar ataxia (SCAR12). So far, this association was not confirmed in additional families. Affected patients presented with childhood-onset cerebellar ataxia, generalized tonic–clonic epilepsy, intellectual disability, and prominent spasticity.^3^ Compared with patients with WOREE syndrome, patients with SCAR12 phenotype have a less severe developmental delay: all were able to walk (4/4; 100%), 3 of 4 were able to speak, and no premature death was reported. When performed, brain MRI showed mild cerebellar hypoplasia (2/2; 100%) in affected patients, a feature not observed in our cohort. Although epilepsy was a constant feature in SCAR12 patients, seizures were more often responsive to antiepileptic drugs (2/4; 50%). Eye involvement was also present with nystagmus reported in four patients. This form of *WWOX*-related autosomal recessive cerebellar ataxia seems to be a rare genetic condition. The severity of the WOREE phenotype and premature death in a proportion of patients might hide balance problems related to cerebellar dysfunction. Additional cases of *WWOX*-related cerebellar ataxia are needed to confirm its implication in this neurological disorder.

### WOREE phenotype

Soon after the description of the SCAR12 phenotype, *WWOX* was implicated in a recessive form of infantile epileptic encephalopathy in 17 patients from 9 families.^4–9,30^ We are more than doubling the number of cases with 20 affected patients in 18 additional families. The phenotype of our patients is consistent with that of previous reports. We observed a lower rate of consanguinity in our cohort in accordance with the higher proportion of compound heterozygous genotypes described in this study. These genotypes might have been previously missed when array-CGH or high-throughput sequencing were performed independently. The implementation of CNV prediction from exome sequence data facilitates the identification of patients with *WWOX* genotypes composed of both SNV and CNV.

One fetus with brain anomalies and possible intrauterine seizures was reported in 2015 (ref. ^7^). Decreased fetal movements were occasionally reported in our series (3/20) whereas increased nuchal translucency was the most frequent sign during pregnancy in previous reports (4/17). Hands and feet contractures were reported in 5 patients in our cohort and in 3 patients from the literature^4–6^ in the prenatal or neonatal period. Decreased fetal movements, increased nuchal translucency, and hands or feet anomalies could be antenatal signs of the disease but are neither specific nor frequent clinical features.

**Fig. 2:**
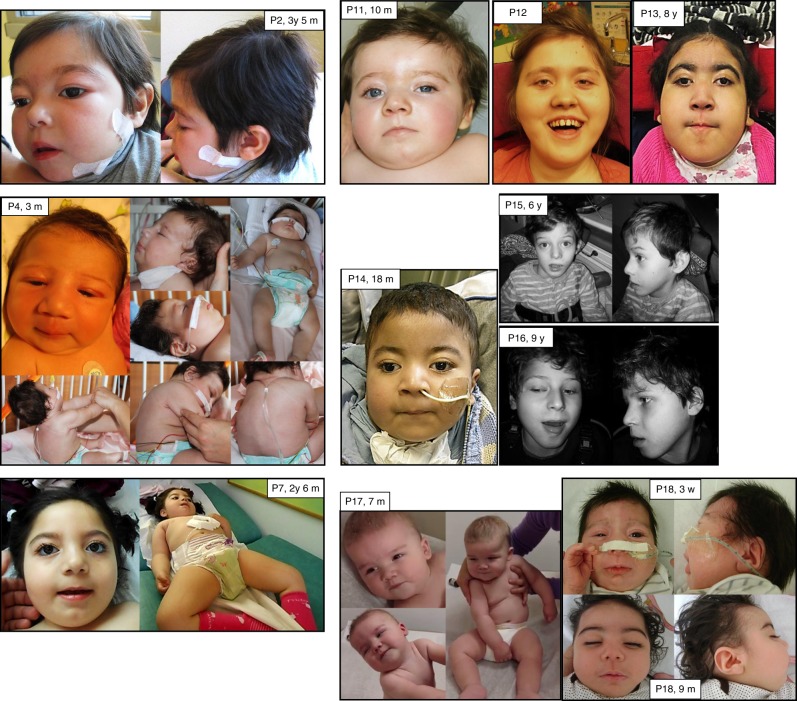
**Photographs of individuals with** ***WWOX*-related epileptic encephalopathy (WOREE) syndrome.** Note major axial hypotonia (P4 and P17); hypotonic facial appearance (all patients); round face, full cheeks, and short neck (P2, P4, P7, P11, P12, P13, P14, P17, and P18).

By contrast to the literature, growth retardation (6%) and microcephaly (20%) were not prominent features in our patients who presented with normal low mean height (–1.1 SD) and mean OFC (–1.4 SD). Dysmorphism was reported in more than half of the patients. The most striking features were a round face with full cheeks, a short neck, and a hypotonic facial appearance (Fig. 2). In patients P15 and P16, at later age, facial hypotonia was still present but the face became more triangular. Additional medical problems were scoliosis or kyphosis of early onset (65%), which might guide the diagnosis, as well as feeding troubles requiring feeding tube in 70% of patients and respiratory problems (40%), as seen in other infantile encephalopathies.

**Fig. 3:**
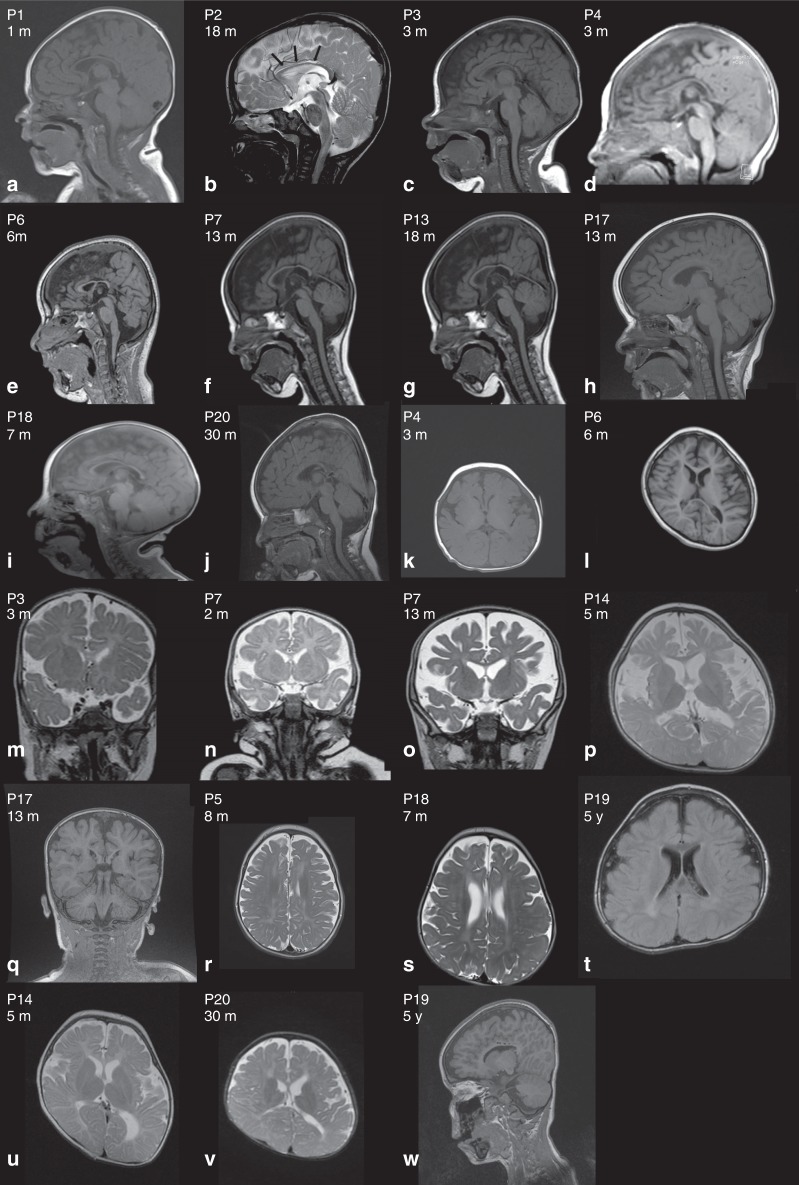
**Brain magnetic resonance image (MRI) of individuals with** ***WWOX*-related epileptic encephalopathy (WOREE) syndrome.** **a**–**j** Hypoplastic corpus callosum on sagittal planes. **k**–**q** Cerebral atrophy and enlarged subarachnoid spaces on axial planes (P4, P6, P14) and coronal planes (P3, P7, P17). **r**–**t** Symmetric white matter hypersignal on axial T2. **u**–**v** Plagiocephaly and asymmetric lateral ventricle on axial T2 planes. **w** P19 sagittal T1 circular lesions (hyposignal) of the medial part of the corpus callosum. *m* month, *P* patient, *y* year old.

All patients had severe developmental delay. None of them were able to sit, speak, or walk. Neurologic examination showed axial hypotonia and distal hypertonia in most of the patients. Dystonic movements were occasionally observed. As reported in the literature, all patients had early-onset epilepsy (100%) with daily seizures and drug resistance observed in most of the cases (95%). There was no major susceptibility to febrile seizures. West syndrome characterized by spasms and hypsarrhythmia recorded on EEG was reported in 26% of patients. Focal, generalized, and combined seizures were seen. Tonic, clonic, tonic–clonic, and myoclonic seizures as well as infantile spasms and absences were described. Thus epilepsy was very severe, of early onset and with variable seizure mainfestions. As previously described, visual impairment seems to be part of the clinical spectrum of WOREE syndrome. Most of the affected patients had no eye contact (75%). Around half of them presented with altered fundus oculi (47%) and/or electrodiagnostic tests (45%) suggestive of optic atrophy and/or retinal dystrophy. Early ophthalmological examination therefore may give diagnostic clues in patients with early-onset seizures and developmental delay. Brain MRI (Fig. 3) was abnormal in 80% of patients mainly showing corpus callosum hypoplasia (75%) and cerebral atrophy (55%). We confirmed in our cohort that premature death is a feature of WOREE syndrome occurring in eight patients (40%) with a mean age at death of 40 months. The severity of epilepsy and neurological impairment may be linked to early death in affected patients.

Main clinical signs in previously published patients with WOREE are summarized in Supplemental Table 4. From this review we can conclude that WOREE is a very severe epileptic encephalopathy characterized by poor spontaneous mobility, absence of language development and acquisition of walking, early-onset drug-resistant seizures, ophthalmological involvement, and a high likelihood of premature death. Dysmorphic features (round hypotonic face, full cheeks, and short neck) and spine deformity may be early associated signs that may support the diagnosis.

### Phenotype/genotype correlations

Phenotype/genotype correlations were recently suggested for *WWOX*-related neurodevelopmental disorders^5,7^ with a classification of *WWOX* genotypes into three groups. According to the tentative classification, patients carrying two predicted null alleles were more likely to present with the most severe WOREE phenotype whereas hypomorphic genotypes with two missense variants would instead result in spinocerebellar ataxia (SCAR12). The phenotype of patients carrying a null allele and a missense pathogenic variation would be intermediate.^5^ Our large study provides a reappraisal of these preliminary correlations.

The most severe clinical presentation seems to be associated with genotypes consisting of early premature stop codons corresponding to *WWOX* full knockdown. These genotypes are observed in seven patients: homozygous deletion of exon 1 to 4 in P10, homozygous stop codons p.(Arg54*) and p.(Trp44*),^4,30^ homozygous deletion of the first six exons.^7^ Premature death before 2 years occurred in 5/7 cases and brain anomalies were detected prenatally in one family. Furthermore, premature death has never been described in patients with missense pathogenic variant–only genotype (Supplemental Tables 1 and 3). The precise characterization of missense variants and CNVs at the protein level with functional studies may give some clues about how genotypes could explain different clinical presentations, especially regarding SCAR12/WOREE phenotypes.

In conclusion, germline pathogenic variants in *WWOX* are clearly associated with a severe early-onset epileptic encephalopathy called WOREE syndrome and we report here on the largest cohort of individuals with this neurological disorder. Implication of constitutional *WWOX* variants in DSD and SCA still needs replication studies.

## Electronic supplementary material

Supplementary Table 1

Supplementary Table 2

Supplementary Table 3

Supplementary Table 4
