# Correction: The phenotypic spectrum of *WWOX*-related disorders: 20 additional cases of WOREE syndrome and review of the literature

**DOI:** 10.1038/s41436-019-0460-y

**Published:** 2019-02-20

**Authors:** Juliette Piard, Lara Hawkes, Mathieu Milh, Laurent Villard, Renato Borgatti, Romina Romaniello, Melanie Fradin, Yline Capri, Delphine Héron, Marie-Christine Nougues, Caroline Nava, Oana Tarta Arsene, Debbie Shears, John Taylor, Alistair Pagnamenta, Jenny C Taylor, Yoshimi Sogawa, Diana Johnson, Helen Firth, Pradeep Vasudevan, Gabriela Jones, Marie-Ange Nguyen-Morel, Tiffany Busa, Agathe Roubertie, Myrthe van den Born, Elise Brischoux-Boucher, Michel Koenig, Cyril Mignot, DDD Study, Usha Kini, Christophe Philippe

**Affiliations:** 1 Centre de Génétique Humaine, Université de Franche-Comté, CHU Besançon, Besançon, France; 2 Oxford Centre for Genomic Medicine, Oxford University Hospitals NHS Foundation Trust, Oxford, UK; 3 Aix Marseille Univ, Inserm, MMG, Marseille, France; 4 Pediatric Neurology, La Timone Children’s Hospital, AP-HM, Marseille, France; 5 Medical Genetics, La Timone Children’s Hospital, AP-HM, Marseille, France; 6 Neuropsychiatry and Neurorehabilitation Unit, Scientific Institute IRCCS Eugenio Medea, Bosisio Parini, Italy; 7 Service de Génétique, CLAD Ouest, CHU Rennes, Rennes, France; 8 Département de Génétique, Hôpital Robert Debré, APHP Paris, Paris, France; 9 APHP, Département de Génétique, Centre de Référence Déficiences Intellectuelles de Causes Rares, Groupe Hospitalier Pitié Salpêtrière et GHUEP Hôpital Trousseau, Sorbonne Université, GRC “Déficience Intellectuelle et Autisme”, Paris, France; 10 Neuropédiatrie et Unité d’électrophysiologie clinique, Centre de Référence des Maladies Neuromusculaires de l’EST parisien et DHU I2B, Hôpital d’Enfants Armand Trousseau, Paris, France; 11 Département de Génétique, Sorbonne Universités, Institut du Cerveau et de la Moelle épinière, ICM, Inserm U1127, CNRS UMR 7225, APHP, Hôpital de la Pitié Salpêtrière, Paris, France; 12 Pediatric Neurology Clinic, “Alexandru Obregia” Clinical Psychiatry Hospital, “Carol Davila” University of Medicine, Bucharest, Romania; 13 Oxford Medical Genetics Laboratories, Oxford University Hospitals NHS Foundation Trust, Churchill Hospital, Oxford, UK; 14 NIHR Oxford Biomedical Research Centre, Wellcome Centre for Human Genetics, University of Oxford, Oxford, UK; 15 Division of Pediatric Neurology, Children’s Hospital of Pittsburgh of UPMC, Pittsburgh, PA USA; 16 Department of Clinical Genetics, Sheffield Children’s NHS Trust, Sheffield, United Kingdom; 17 Department of Clinical Genetics, Cambridge University Hospitals NHS Foundation Trust, Cambridge, UK; 18 Wellcome Trust Sanger Institute, Wellcome Genome Campus, Hinxton, UK; 19 Clinical Genetics Department, University Hospitals Leicester NHS Trust, Leicester, UK; 20 Service de Neurologie pédiatrique, Hopital Couple Enfant, CHU Grenoble Alpes, Grenoble, France; 21 Département de Neuropédiatrie, Centre Hospitalier Universitaire de Montpellier, INSERM U 1051, Institut des Neurosciences de Montpellier, Montpellier, France; 22 Department for Clinical Genetics, Erasmus MC, Rotterdam, Netherlands; 23 EA7402 Institut Universitaire de Recherche Clinique, and Laboratoire de Génétique Moléculaire, CHU and Université de Montpellier, Montpellier, France; 24 Laboratoire de génétique, Innovations en diagnostic génomique des maladies rares, Plateau Technique de Biologie, CHU Dijon, Dijon, France; 25 INSERM 1231, LNC UMR1231 GAD, Burgundy University, Dijon, France

Correction to: *Genetics in Medicine*; published online 25 October 2018; 10.1038/s41436-018-0339-3

The article has been corrected to account for one patient being investigated through genome sequencing rather than exome sequencing as originally published; thus amendments to the Abstract and Methods have been made as well as addition of the relevant authors and acknowledgment.

